# Identification of Persistent and Resurgent Sodium Currents in Spiral Ganglion Neurons Cultured from the Mouse Cochlea

**DOI:** 10.1523/ENEURO.0303-17.2017

**Published:** 2017-11-14

**Authors:** Lorcan Browne, Katie E. Smith, Daniel J. Jagger

**Affiliations:** UCL Ear Institute, University College London, London WC1X 8EE, United Kingdom

**Keywords:** cochlea, hearing, ion channels, persistent current, resurgent current, spiral ganglion

## Abstract

In spiral ganglion neurons (SGNs), the afferent single units of the auditory nerve, high spontaneous and evoked firing rates ensure preservation of the temporal code describing the key features of incoming sound. During postnatal development, the spatiotemporal distribution of ion channel subtypes contributes to the maturation of action potential generation in SGNs, and to their ability to generate spike patterns that follow rapidly changing inputs. Here we describe tetrodotoxin (TTX)-sensitive Na^+^ currents in SGNs cultured from mice, whose properties may support this fast spiking behavior. A subthreshold persistent Na^+^ current (I_NaP_) and a resurgent Na^+^ current (I_NaR_) both emerged prior to the onset of hearing and became more prevalent as hearing matured. Navβ4 subunits, which are proposed to play a key role in mediating I_NaR_ elsewhere in the nervous system, were immunolocalized to the first heminode where spikes are generated in the auditory nerve, and to perisomatic nodes of Ranvier. ATX-II, a sea anemone toxin that slows classical Na^+^ channel inactivation selectively, enhanced I_NaP_ five-fold and I_NaR_ three-fold in voltage clamp recordings. In rapidly-adapting SGNs under current clamp, ATX-II increased the likelihood of firing additional action potentials. The data identify I_NaP_ and I_NaR_ as novel regulators of excitability in SGNs, and consistent with their roles in other neuronal types, we suggest that these nonclassical Na^+^ currents may contribute to the control of refractoriness in the auditory nerve.

## Significance Statement

Neurons in the auditory nerve are renowned for their ability to fire action potentials at high rates. This is essential for the brain’s normal coding of acoustic signals, and in humans this information is important for deciphering speech, and for determining the frequency and loudness of sounds. Here, we describe mechanisms that may contribute to this rapid spiking. Nonclassical Na^+^ currents increase the excitability and decrease the refractoriness of spiral ganglion neurons (SGNs), enabling them to fire spikes at high rates for prolonged periods. Knowing how these currents contribute to normal auditory nerve function may improve our understanding of peripheral auditory neuropathies, and identify novel drug targets for the treatment of conditions causing hearing loss.

## Introduction

The auditory nerve connects the cochlea to the cochlear nucleus, and so acts as the gateway to the hearing brain. Individual spiral ganglion neurons (SGNs) convey the requisite information describing the essential properties of incoming sound, and so preserve the timing cues contributing to coding of frequency, localization and loudness within higher centers. SGNs translate neurotransmitter release from mechano-sensory hair cells into action potentials, which are propagated along their neurites forming the main body of the auditory nerve. In the functionally mature cochlea, each inner hair cell (IHC) contacts 5-30 unbranched nerve endings of type I SGNs (Rusznák and Szucs, 2009; Meyer and Moser, 2010; Nayagam et al., 2011; Heil and Peterson, 2015).

Auditory nerve recordings from a number of mammalian species have revealed that SGNs can fire spontaneously in the absence of sound, and that they increase their firing rate in response to presented tones, with the initial rate being proportional to the sound intensity (Heil and Peterson, 2015). Recordings from individual SGNs in mice have demonstrated peak discharge rates >1 kHz (Taberner and Liberman, 2005), which then adapt to steady state levels >200 Hz, suggesting these neurons employ specialized mechanisms that support repetitive rapid spiking and ensure extremely short refractory periods. Patch clamp recordings and immuno-labeling experiments have suggested that the expression of numerous ion channel subtypes contributes to shaping the firing properties of SGNs, in mice and rats (Jagger and Housley, 2002, 2003; Hossain et al., 2005; Wang et al., 2013; Crozier and Davis, 2014; Smith et al., 2015; Kim and Rutherford, 2016; Wright et al., 2016), and in guinea pigs (Santos-Sacchi, 1993; Szabó et al., 2002). Following hearing onset, increases in firing rates and shortening of the refractory period are observed (Wu et al., 2016). This maturation of firing properties may in part be explained by the recruitment of voltage-gated Na^+^ channels and K^+^ channels to distinct subcellular microdomains (Kim and Rutherford, 2016). The outcome is a microsecond postsynaptic precision in functionally mature SGNs that preserves the timing of presynaptic neurotransmitter release (Rutherford et al., 2012).

Elsewhere in the nervous system, rapid spiking behavior is often associated with mechanisms that avoid long-lasting Na^+^ channel inactivation in preference for alternative states that enable shorter refractory periods. “Persistent” Na^+^ currents (I_NaP_) activate at membrane potentials just below the spike threshold, and can amplify the response to synaptic inputs and enhance the neuronal capability to fire repetitively (Crill, 1996; Stafstrom, 2007). “Resurgent” Na^+^ currents (I_NaR_) activate transiently during an action potential repolarization following the initial strong depolarization, raising the likelihood of a second spike (Bean, 2007; Theile and Cummins, 2011; Lewis and Raman, 2014). Here we provide the first description of comparable tetrodotoxin (TTX)-sensitive currents in SGNs cultured from mice. Nav1.6 α-subunits and regulatory Navβ4 subunits are suggested to mediate these currents in other neuronal types (Theile and Cummins, 2011; Lewis and Raman, 2014), and in fixed cochlear sections these subunits were both localized within domains that regulate spike generation and propagation in the auditory nerve. The currents emerged at the end of the first postnatal week, and increased their prevalence around the onset of hearing. Pharmacological modulation of I_NaP_ and I_NaR_ altered the excitability and firing behavior of SGNs, suggesting these anti-refractory mechanisms could play key roles in the regulation of spike firing in the mature auditory nerve.

## Materials and Methods

### Animals

C57BL/6 mice of either sex were bred within an in-house facility, and were killed by cervical dislocation and subsequent decapitation. All animal work conformed to United Kingdom legislation outlined in the Animals (Scientific Procedures) Act 1986.

### SGN culture

The cochleae of mice of either sex between postnatal day 0 (P0) and P90 were removed and the bony modioli isolated. The tissue was digested in 0.25% trypsin at 37°C for 30 min. Growth medium (DMEM containing 10% FCS, 10 mM HEPES, and 1% penicillin/streptomycin) was added and the tissue was gently triturated. Cells were pelleted by gentle centrifugation (400 × *g*, 10 min), resuspended in growth medium, and then plated onto glass coverslips pretreated with poly-L-lysine (50 µg/ml). Coverslips were incubated in a humidified incubator at 37°C, 5% CO_2_, for 1 h to allow the dissociated SGNs to adhere, and then growth medium supplemented with 10 ng/ml brain-derived neurotrophic factor (BDNF) was added. Patch clamp recordings were performed following 1–2 d *in vitro*. Chemicals were obtained from Sigma-Aldrich unless stated otherwise.

### SGN electrophysiology

Recordings were conducted in the whole-cell configuration using an Axopatch 200B patch clamp amplifier (Molecular Devices) and a Digidata board (Molecular Devices) under the control of pClamp software (version 8, Molecular Devices). During initial recordings cultures were superfused with artificial perilymph (containing 145 mM NaCl, 4 mM KCl, 1 mM MgCl_2_, 1.3 mM CaCl_2_, 10 mM HEPES, and 5 mM glucose; pH adjusted to 7.3 with NaOH) under the control of a peristaltic pump. Patch pipettes were pulled from capillary glass (GC120TF; Harvard Apparatus) using a vertical puller (PP-830; Narishige). Pipettes had resistances of 3-4 MΩ when filled with an intracellular solution containing 130 mM K-gluconate, 5 mM KCl, 2 mM MgATP, 2 mM Na_2_ATP, 0.3 mM Na_3_GTP, 10 mM Na_2_-phosphocreatine, 1 mM EGTA, and 10 mM HEPES; pH adjusted to 7.2 with KOH. In some experiments, macroscopic Na^+^ currents (I_Na_) were recorded in isolation using an extracellular solution supplemented with blockers of K^+^ currents (I_K_), Ca^2+^ currents (I_Ca_), and hyperpolarization-activated cation currents (I_h_), containing 145 mM NaCl, 4 mM KCl, 1 mM MgCl_2_, 1.3 mM CaCl_2_, 0.3 mM CdCl_2_, 2 mM CsCl, 10 mM HEPES, and 5 mM glucose; pH adjusted to 7.3 with NaOH. For these recordings the intracellular solution contained 120 mM CsF, 25 mM TEA-Cl, 2 mM MgATP, 2 mM Na_2_ATP, 0.3 mM Na_3_GTP, 2.5 mM Na_2_-phosphocreatine, 1 mM EGTA, and 10 mM HEPES; pH adjusted to 7.2 with TEA-OH. TTX-sensitive currents were obtained by digital subtraction of averaged (2×) recordings in the presence of TTX from averaged recordings in control conditions. The TTX-sensitive current is denoted as “difference current” in the text. Consistent with the existing literature, maximum persistent Na^+^ current (I_NaP_) and resurgent Na^+^ current (I_NaR_) amplitudes in individual SGNs are expressed as a percentage of the maximum transient Na^+^ current (I_NaT_) measured in the same cell. Liquid junction potentials were calculated and subtracted off-line. Series resistance compensation was applied routinely at 70%. All recordings were performed at room temperature (22-24°C). Current clamp and voltage clamp protocols are described in the results section and in figure legends. Electrophysiological data were analyzed off-line using Clampfit (version 10; Molecular Devices), Igor Pro (version 6.36, Wavemetrics), and OriginPro 2016 (OriginLab). The voltage dependence of activation and inactivation were calculated by fitting conductance-voltage data with the Boltzmann function, G_norm_ = G_min_ + (G_max_ − G_min_)/(1 + exp((V_1/2_ − V_m_)/*k*)), where G is conductance, V_1/2_ is the voltage of half-maximal activation or inactivation, V_m_ is the membrane potential, and *k* is the slope factor.

### Drug stocks and application

Dendrotoxin-K (DTX-K), TTX, 4,9-anhydro-TTX (4,9-ah-TTX), and sea anemone (*Anemonia sulcata*) toxin ATX-II were all obtained from Alomone Labs. Toxins were prepared as stock solutions in water and stored at −20°C, before dilution in extracellular solution for patch clamp recordings.

### Antibodies

The mouse monoclonal neuronal class III β-tubulin antibody (TUJ1; subtype IgG_2a_; Covance, Princeton) was used as a neuronal marker. Nav1.6 subunits were detected using a rabbit polyclonal anti-Nav1.6 antibody (ASC-009; Alomone Labs). Mouse monoclonal antibodies against Navβ4 (subtype IgG_1_; clone N168/6), Kv1.2 (subtype IgG_2b_; clone K14/16) and Caspr (subtype IgG_1_; clone K65/35) were obtained from the UC Davis/NIH NeuroMab Facility. Mouse monoclonal antibodies were detected by the application of isotype-specific secondary antibodies: Alexa Fluor 488 goat anti-mouse IgG_2b_, Alexa Fluor 555 goat anti-mouse IgG_1_ and Alexa Fluor 633 goat anti-mouse IgG_2a_ (Life Technologies). Alexa Fluor conjugated goat or donkey anti-rabbit secondary antibodies were used to detect the rabbit anti-Nav1.6 antibody (Life Technologies).

### Immunofluorescence

Whole cochleae from P21 mice were fixed in either 2% (for Navβ4 labeling) or 4% paraformaldehyde in PBS for 40 min at room temperature. The fixed cochleae were washed several times in PBS, and then decalcified in 4% EDTA for 48 h, at 4°C. The semicircular canals were removed and the remaining otic capsules were mounted in 4% low-melting point agarose and sectioned on a vibratome (1000 plus system, Intracel) at 150- to 200-µm intervals. Antibody labeling was performed on mid-modiolar sections. Following an initial block and permeabilization (10% normal goat serum and 0.2% Triton X-100 in PBS) for 1 h at room temperature, the sections were further incubated in Mouse Ig Blocking Reagent (Vector Laboratories), for 1 h at room temperature, to minimize the background fluorescence of the mouse tissue. All primary antibodies were diluted 1:100, with the exception of TUJ1 (1:500), in lysine blocking solution (0.1 M lysine and 0.2% Triton X-100 in PBS). In control experiments the primary antibodies were omitted. For Nav1.6 labeling, primary antibodies were incubated for 3.5 h at room temperature. For anti-Navβ4 experiments, the primary antibodies were incubated overnight at 4˚C. Following several washes in PBS, the sections were incubated in Alexa Fluor secondary antibodies diluted 1:400 in lysine blocking solution at room temperature for 1 h. After a final set of washes in PBS, sections were mounted in Vectashield containing DAPI (Vector Laboratories). Images were acquired using a 20× air immersion objective (N.A. 0.75) or a 63× water immersion objective (N.A. 1.2) of an LSM510 confocal microscope (Carl Zeiss Microscopy) equipped with 405, 488, 543, and 633 nm lasers; 505-530 and 560-615 nm bandpass filters were used to collect Alexa Fluor 488 and 555 emissions, respectively. A 650 nm long-pass filter was used to collect Alexa Fluor 633 emission. DAPI emission was collected using a 420–480 nm bandpass filter. Multi-channel z-stacks were acquired using sequential scanning, with a frame average of 4 and a *z*-step size of 2.3 µm (20×) or 1.4 µm (63×). Images showing Nav channel immunofluorescence are maximum intensity *z*-projections of two adjacent sections (spiral ganglion region), or four adjacent sections (hair cell region).

### Experimental design and statistical analysis

For group data the values given in the text are mean data ± the standard error of the mean. In experiments with low *n* values, representative measurements or traces are shown alongside the number of observations made. Where appropriate, normality of datasets was assessed using the Shapiro-Wilk test, and an *F* test was used to assess if the variances were equal between groups. Where data fulfilled the requirements for parametric analysis, statistical significance was determined using Student’s *t* test. A Mann–Whitney *U* test or Wilcoxon signed-rank test analyzed nonparametric data where appropriate. ANOVA determined differences between three or more means. To test for within and between group effects, analysis was conducted using the standard Bonferroni posthoc test. Alternatively, when it was necessary to run multiple tests in the absence of an ANOVA analysis, multiple *t* tests or Mann–Whitney *U* tests were performed manually and Bonferroni correction was used to avoid possible type-1 errors resulting from multiple comparisons. All *p* values were reported relative to a significance level (α) of 0.05.

## Results

### Multiple TTX-sensitive Na^+^ currents in SGNs around hearing onset

**Figure 1.**
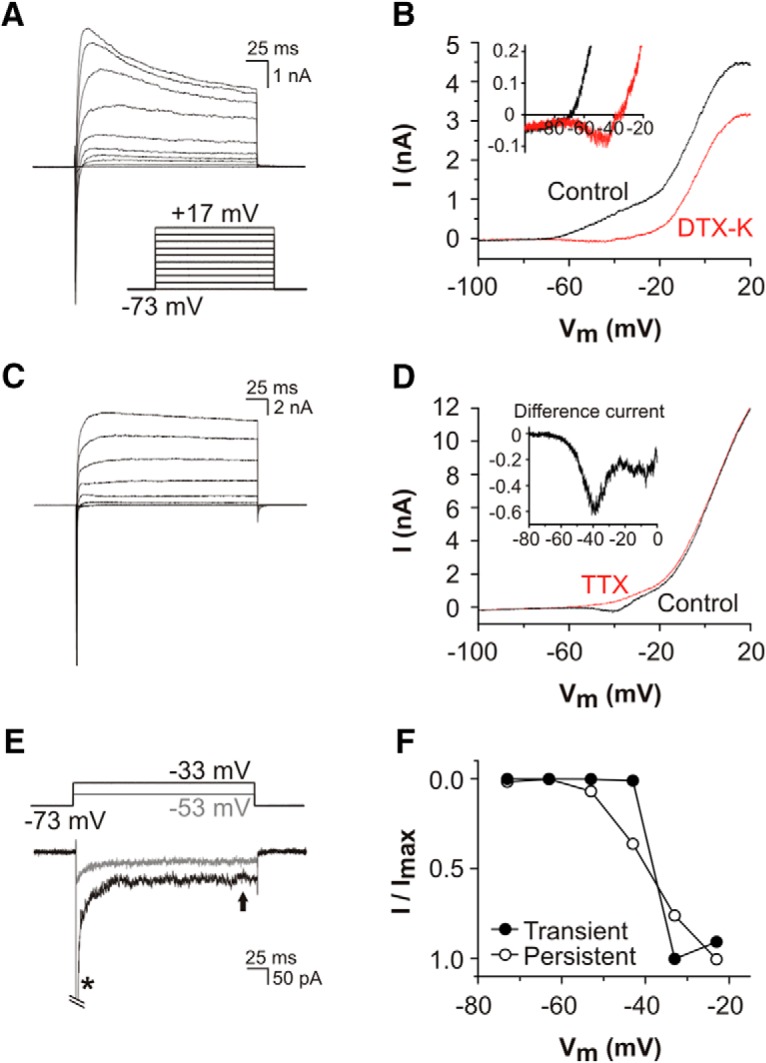
TTX-sensitive transient and persistent Na^+^ currents identified in SGNs cultured from hearing mice. ***A***, Current responses recorded using K^+^-filled patch electrodes, from a representative rapidly-adapting P14 SGN during 200-ms voltage steps. A transient inward Na^+^ current (I_NaT_) was followed by LVA and HVA outward K^+^ currents. ***B***, Current response from the same cell during a voltage ramp (between −143 and +47 mV, duration 400 ms) before (control) and after bath application of 100 nM DTX-K. DTX-K blocked the LVA K^+^ current, revealing a small inward current activated at potentials positive to −65 mV (detailed in the inset). ***C***, Current responses recorded using K^+^-filled patch electrodes, from a representative nonadapting P14 SGN during 200-ms voltage steps (protocol described in ***A***). Activation of I_NaT_ was followed by activation of HVA K^+^ currents. LVA K^+^ currents were absent from this cell. ***D***, Current responses from the same SGN, during a voltage ramp before (control) and after bath application of 100 nM TTX. A TTX-sensitive inward current activated at potentials positive to −65 mV (difference current, detailed in the inset). ***E***, TTX-subtracted currents recorded from the SGN in ***D***, during 200-ms depolarizing voltage steps. A voltage step to −53 mV activated a small persistent inward current (I_NaP_), whereas the voltage step to −33 mV activated a large I_NaT_ (*, peak current cropped for clarity) and a small I_NaP_. ***F***, Comparison of the voltage dependence of TTX-sensitive I_NaT_ and I_NaP_ (measured at the arrow in ***E***) in this SGN.

Initial whole-cell voltage clamp recordings were conducted on SGNs cultured from mice after the onset of hearing (P12-P14; Fig. 1), and these revealed families of currents that resembled those in studies using comparable preparations (Santos-Sacchi, 1993; Szabó et al., 2002; Smith et al., 2015). At this developmental stage SGNs can be categorized broadly as rapidly-adapting (firing one to two action potentials in response to sustained current injection), slowly-adapting (three to seven action potentials) or nonadapting (tonic firing). In experiments here using K^+^-containing pipette solutions, depolarizing voltage steps activated transient inward Na^+^ currents (I_NaT_) and sustained outward K^+^ currents. In rapidly-adapting SGNs (Fig. 1*A*), the outward K^+^ currents had a distinct low voltage-activated (LVA) component in addition to a high voltage-activated (HVA) component. Voltage ramps (between −143 and +47 mV, duration 400 ms) revealed the LVA current activated at potentials positive to −65 mV, and the HVA component activated above −20 mV (Fig. 1*B*). Selective block of the LVA current by bath-applied DTX-K revealed an underlying inward current that activated at potentials positive to −70 mV and had a maximum amplitude around −40 mV (Fig. 1*B*, inset).

In nonadapting SGNs there is little measureable LVA current under control conditions (Fig. 1*C*), and during voltage ramps in such cells there was an inward current that activated around the same voltage range as described above (Fig. 1*D*). This current was blocked by a low concentration of bath-applied TTX (100 nM). The digitally subtracted TTX-sensitive current had a peak amplitude around −40 mV (measured as 0.6 nA for the example shown in Fig. 1*D*, inset). This difference current resembled voltage ramp-activated persistent Na^+^ currents (I_NaP_), that have been described in a wide variety of neurons (Crill, 1996; Stafstrom, 2007). To further examine this current a voltage step protocol was applied (Fig. 1*E*). From a holding potential of −73 mV, a depolarizing step to −53 mV activated I_NaP_ only, whereas a depolarizing step to −33 mV activated both I_NaT_ and I_NaP_. Comparison of the relative activation ranges of these currents confirmed that I_NaP_ could be elicited at potentials ∼20 mV more negative than I_NaT_ (Fig. 1*F*).

**Figure 2.**
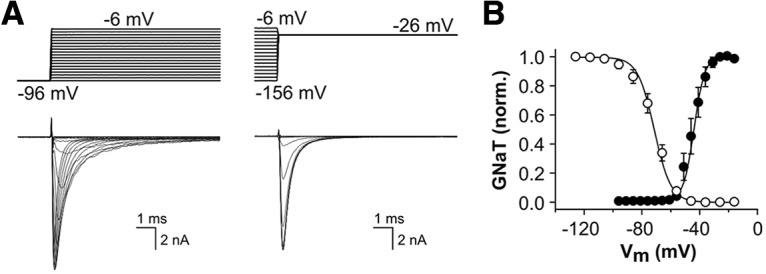
Isolation of transient Na^+^ currents in SGNs cultured from hearing mice. Isolated Na^+^ currents were recorded using Cs^+^-containing patch electrodes and external medium supplemented with channel blockers (see Materials and Methods). ***A***, TTX-sensitive I_NaT_ recorded in a representative P14 SGN. The voltage clamp protocols shown in the upper panels were used to determine the voltage dependence of activation (left; detail of 20-ms test potentials between −96 and – 6 mV, from a holding potential −96 mV) and steady-state inactivation (right; detail of 20-ms test potentials at −26 mV, following 200-ms conditioning potentials between −156 and −6 mV). ***B***, Normalized peak conductance-voltage plots showing activation (filled circles) and inactivation (open circles) of the channels underlying TTX-sensitive I_NaT_ in SGNs cultured from P12-P14 mice (*n* = 10).

To examine the characteristics of whole-cell Nav currents in isolation, K^+^ currents (I_K_), hyperpolarization-activated currents (I_h_) and Ca^2+^ currents (I_Ca_) were minimized (using Cs^+^-based intracellular pipette solution, and an external solution supplemented with blockers of I_K_, I_h_ and I_Ca_ – see Materials and Methods). Under these conditions, the voltage dependence of activation and inactivation for the channels underlying the TTX-sensitive I_NaT_ were determined in SGNs cultured from hearing mice (P12-P14; Fig. 2*A*,*B*). The voltage of half-maximal activation was −44.21 ± 2.05 mV, with a slope value (*k*) of 2.86 ± 0.37 mV; the voltage of half-maximal inactivation was −72.16 ± 2.03 mV with a slope value of 5.39 ± 0.25 mV (*n* = 10). The activation threshold of I_NaT_, around −60 mV, was similar to that described for transient Na^+^ currents in SGNs from adult guinea pigs (Santos-Sacchi, 1993), but the voltage of half-maximal inactivation measured here was slightly hyperpolarized compared to the quoted values of −63 and −66 mV for guinea pig SGNs.

**Figure 3.**
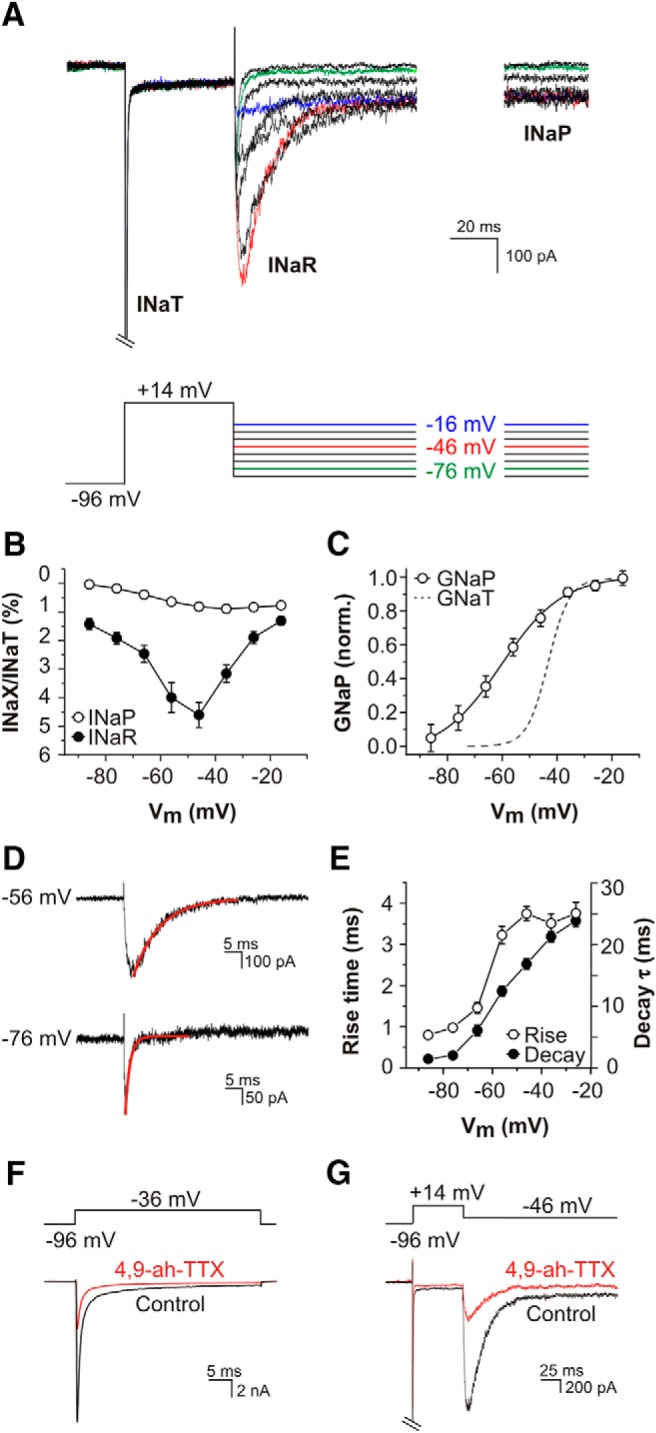
Kinetics, voltage dependence, and pharmacology of resurgent and persistent Na^+^ currents in SGNs. ***A***, Family of TTX-sensitive difference currents evoked by 50-ms step depolarization to +14 mV, followed by 200-ms step repolarization to potentials between −16 and −86 mV. Steps to particular potentials are denoted by the colored traces in the voltage protocol. I_NaT_ was activated during the steps to +14 mV (cropped for clarity). I_NaR_ activated rapidly on repolarization and then decayed, revealing the noninactivating I_NaP_ at the end of the test steps. ***B***, Current−voltage (*I-V*) relationships for maximum TTX-sensitive I_NaR_ (filled circles) and steady-state I_NaP_ (open circles; measured between 160 and 190 ms after the onset of the repolarization steps). I_NaR_ and I_NaP_ are shown as a percentage of maximum I_NaT_ recorded in the same cell (*n* = 30; P12-P14). ***C***, Normalized steady-state conductance-voltage plot for the channels underlying I_NaP_ (open circles), fitted with a Boltzmann function (*n* = 23; P12-P14). For comparison, the peak conductance-voltage plot for I_NaT_ is also shown (discontinuous line, from Fig. 2*B*). ***D***, The kinetics of activation and decay of I_NaR_ varied with repolarization potential. During the protocol used in ***A***, repolarization to −76 mV elicited a current with shorter rise time and decay (exponential fit overlaid in red) compared to that elicited by repolarization to −56 mV. ***E***, Mean onset (time to peak) and offset (decay time constant) kinetics of I_NaR_ in 19 SGNs (P12-P14). ***F***, ***G***, 4,9-ah-TTX, a toxin specific for Nav1.6 subunits blocks I_NaT_, I_NaR_ and I_NaP_. ***F***, A depolarizing voltage step to −36 mV activated I_NaT_ (black trace), which was blocked >70% by bath applied 100 nM 4,9-ah-TTX (red). ***G***, A repolarization from +14 to −46 mV activated I_NaR_ and I_NaP_ (black trace), which were both blocked ∼70% by bath applied 100 nM 4,9-ah-TTX (red).

Following our unexpected observation of I_NaP_, we next explored the possibility of an additional nonclassical Na^+^ current in SGNs, namely the resurgent Na^+^ current (I_NaR_). I_NaR_ is prevalent in certain fast-spiking neurons in the CNS, and is suggested to be mediated by an intracellular blocking peptide that plugs the pore of the Na^+^ channel α-subunit (Bean, 2007; Lewis and Raman, 2014). Recovery from this alternative mode of inactivation is faster than recovery from classical inactivation, allowing I_NaR_ to flow during the repolarization phase of an action potential. The combination of this depolarizing current and the pool of available channels provided by rapid relief from inactivation promotes firing of a second spike (Bean, 2007). To activate both I_NaP_ and I_NaR_ we employed a voltage clamp protocol used previously to characterize these currents in cerebellar Purkinje neurons (Raman and Bean, 1997). P12-P14 SGNs were subjected to a strong initial depolarization (to +14 mV) to fully activate and inactivate I_NaT_, and were then repolarized to a range o*f* test potentials between −86 and −16 mV (Fig. 3*A*). Repolarization elicited a transient inward I_NaR_ at all test potentials, with a maximum amplitude at −46 mV. By maintaining the repolarizing test potential for >100 ms we could also characterize the voltage dependence of the channels underlying noninactivating I_NaP_ (Fig. 3*A*,*C*). Maximum I_NaR_ was 4.61 ± 0.44% (range 1.25–12.07%; *n* = 30) of maximum I_NaT_. In comparison, maximum I_NaP_ was only 0.89 ± 0.14% (range 0.30-3.39%; *n* = 30) of maximum I_NaT_ (Fig. 3*B*). The conductance-voltage plot for the channels underlying I_NaP_ demonstrated a more hyperpolarized activation (V_1/2_ = −55.84 ± 2.29 mV, *k* = 12.71 ± 2.06 mV; *n* = 23; Fig. 3*C*) in comparison to I_NaT_ (Fig. 2*B*). During each repolarizing step I_NaR_ activated rapidly but deactivated more slowly (Fig. 3*A*,*D*). During the most hyperpolarized test potentials, the time to peak of I_NaR_ was measured below 1 ms, and the decay time constant was around 2 ms. During more depolarized test potentials both the rise time and decay were slower (Fig. 3*E*), suggesting that the inactivation peptide is expelled from the Na^+^ channel α-subunit more rapidly at negative repolarization potentials (Lewis and Raman, 2014).

The expression of Nav1.6 α-subunits has been associated with rapid spontaneous and evoked spiking behavior in a number of neuronal subtypes. Some of its functional characteristics facilitate such behavior, including a relatively hyperpolarized voltage dependence of activation, a resistance to inactivation during trains of action potentials, an expression at high densities at sites of action potential generation, and its prominent involvement in the generation of I_NaP_ and I_NaR_ (O'Brien and Meisler, 2013). Nav1.6 subunit expression has been reported previously in SGNs of mice (Hossain et al., 2005) and rats (Fryatt et al., 2009; Kim and Rutherford, 2016). To investigate the possible contribution of Nav1.6 subunits to the Nav currents in cultured mouse SGNs, we used the TTX metabolite 4,9-ah-TTX, which at 100 nM is a specific blocker of these channels (Rosker et al., 2007). The application of 100 nM 4,9-ah-TTX reduced the amplitude of I_NaT_ activated during depolarizing test potentials by 71.7 ± 3.4% (*n* = 6; Fig. 3*F*), and reduced the amplitude of I_NaR_ and I_NaP_ activated during a repolarizing step to −46 mV by 67.4 ± 4.2 and 70 ± 3.3%, respectively (*n* = 6; Fig. 3*G*). The relative (%) reduction in amplitude of the three currents was not significantly different (ANOVA, *F*_(2,15)_ = 0.34, *p* = 0.71). These observations suggest that Nav1.6 subunits most likely contribute to all three Na^+^ currents in SGNs, and that one population of channels operates in a multi-modal fashion to mediate separate currents under specific circumstances. The residual currents in the presence of 4,9-ah-TTX may represent unblocked Nav1.6 channels (requiring a higher, although less selective, dose of 4,9-ah-TTX), or they may be mediated by subunits not targeted by the toxin. Further functional studies are required to characterize the specific molecular basis of these currents.

### Navβ4 subunits localize to the spike initiator and perisomatic nodes of Ranvier

Nav1.6 α-subunits are most likely modulated by Navβ4 accessory subunits to mediate I_NaR_ in cerebellar Purkinje neurons (Grieco et al., 2005), although other proteins may contribute to the process (Yan et al., 2014; Ransdell et al., 2017). The cytoplasmic tail of the Navβ4 subunit is proposed to enact an open channel block during strong depolarizations, preventing conventional fast inactivation of Nav1.6 subunits, and this blocking peptide then dissociates readily from the α-subunit when the cell is repolarized to enable an inward resurge of current (Theile and Cummins, 2011; Lewis and Raman, 2014). The mechanism of I_NaP_ generation is presently less clear, although it may result from incomplete classical inactivation of Nav subunits (Crill, 1996). Following the electrophysiological identification of I_NaR_ in SGNs and its blockade by a Nav1.6-specific toxin, we next sought to determine if Navβ4 subunits were expressed in the neuronal microdomains associated with action potential generation and propagation within the auditory nerve. For these experiments we used a mouse monoclonal antibody which has been characterized previously (Buffington and Rasband, 2013), and which has been found to be specific for the Navβ4 subunit.

**Figure 4.**
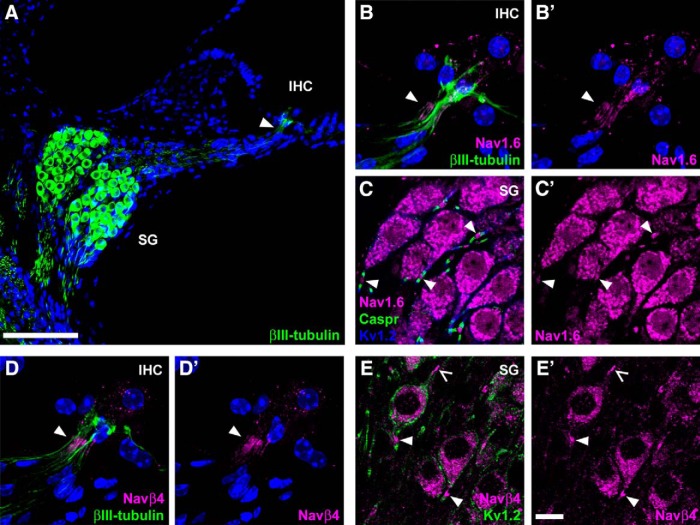
Navβ4 subunits localize to neuronal microdomains associated with spike generation and propagation. ***A***, SGN cell bodies reside in the spiral ganglion (SG), with their peripheral neurites extending toward an IHC. Arrowhead indicates the first heminode region, the site of action potential initiation. Nuclei were labeled using DAPI (blue). Scale bar, 100 µm. ***B–B’***, Immunofluorescence for Nav1.6 subunits localizes to the first heminode (arrowhead) below the IHC. Nuclei were labeled using DAPI (blue). ***C–C’***, Within the SG, Nav1.6 subunits localize to pre- and postsomatic nodes of Ranvier (arrowheads), in addition to strong cytoplasmic expression within the soma of SGNs. Caspr and Kv1.2 antibodies label paranodal and juxtaparanodal regions, respectively. ***D–D’***, Immunofluorescence for Navβ4 subunits localizes to the first heminode (arrowhead). Nuclei were labeled using DAPI (blue). ***E–E’***, Within the SG, Navβ4 subunits localize to pre-somatic (open arrow) and post-somatic (arrowheads) nodes of Ranvier. Scale bar in ***E’***, 10 µm, refers to panels ***B–E***.

βIII-tubulin immunofluorescence demonstrated the bipolar morphology of SGNs within vibratome sections cut from the cochleae of young adult mice (Fig. 4*A*). Their somata reside in the spiral ganglion with their peripheral neurites extending to the sensory IHCs, and their central neurites extending toward the brainstem. Action potentials are generated in the peripheral neurites close to the synapses with IHCs, where Nav and Kv channel subunits are arranged within a heminodal structure (Hossain et al., 2005; Smith et al., 2015; Kim and Rutherford, 2016). Action potentials are propagated along the peripheral neurites via nodes of Ranvier, with pre-somatic and post-somatic nodes of Ranvier either side of the SGN somata. Nav1.6 subunits have been localized previously to the first heminode and nodes of Ranvier in SGNs of mice and rats, and they are proposed to be key contributors to action potential initiation and propagation (Hossain et al., 2005; Kim and Rutherford, 2016). Consistent with these findings, in cochlear sections from P21 mice Nav1.6 immunofluorescence was localized to the first heminode (Fig. 4*B*, arrowhead) and to nodes of Ranvier, including the peri-somatic nodes in the spiral ganglion (Fig. 4*C*, arrowheads). Here, Nav1.6 immunofluorescence was flanked by labeling for the paranodal marker Caspr (Kim and Rutherford, 2016), and by labeling for the juxtaparanodal marker Kv1.2 (Smith et al., 2015). Navβ4 immunofluorescence also localized to the first heminode (Fig. 4*D*, arrowhead) and to nodes within the spiral ganglion (Fig. 4*E*, arrowheads). There was no labeling of these structures in the control experiments where the primary antibodies were omitted (data not shown). The localization of Nav1.6 and Navβ4 immunofluorescence to the same microdomains within SGNs raises the possibility that they interact functionally to mediate I_NaR_.

### Increased prevalence of persistent and resurgent Na^+^ currents by hearing onset

We extended our investigation of I_NaP_ and I_NaR_ to study their first appearance and their subsequent progression during the postnatal period. During this period there is a maturation of spike train properties toward more random firing, and there is a shortening of refractoriness (Wu et al., 2016). In a recent report of neuronal properties in cultured spiral ganglion explants, mature firing behavior was first observed in SGNs derived from the basal (high-frequency coding) region of the cochlea, and subsequently in those from the apical (low-frequency coding) region (Crozier and Davis, 2014). These changes are consistent with the hypothesis that maturation of cellular function in the auditory periphery follows a progression from the base of the cochlea to the apex (Mann and Kelley, 2011).

**Figure 5.**
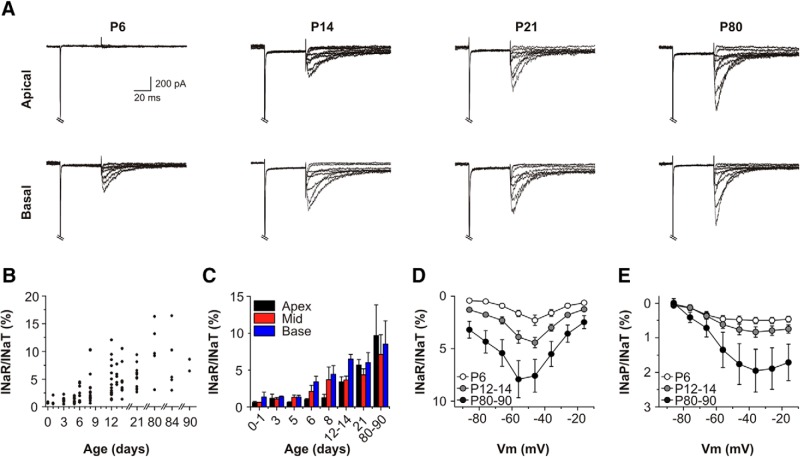
Persistent and resurgent Na^+^ currents have an increased prevalence by hearing onset. ***A***, Representative Na^+^ currents elicited in basal and apical SGNs, at developmental stages before (P6) and after the onset of hearing (P14, P21, P80). TTX-sensitive difference currents were elicited using the voltage protocol in Figure 3*A*. **B**, Scatter plot of I_NaR_/I_NaT_ ratios for individual SGNs at various developmental ages. ***C***, Variations in I_NaR_/I_NaT_ at different tonotopic locations. Individual data from ***B*** has been grouped (P0-P1, P3, P5, P6, P8, P12-P14, P21, and P80-P90) and separated into tonotopic origin (base, mid, and apex). ***D***, Mean I_NaR_/I_NaT_ ratio for all SGNs (basal and apical cells pooled) plotted as a function o*f* test potentials at various developmental ages (P6, *n* = 9; P12-P14, *n* = 30; P80-P90, *n* = 12). ***E***, Mean I_NaP_/I_NaT_ ratio plotted as a function of test potentials, for the same cells as in ***D***.

We examined the relative amplitudes of I_NaP_ and I_NaR_ in the different frequency coding regions of the cochlea during the period from birth up to three months of age (Fig. 5). Spiral ganglia were dissected and divided into basal, mid and apical lengths. Dissociated SGNs from these regions were cultured separately, and whole-cell recordings allowed comparison of the relative contributions of I_NaT_, I_NaP_ and I_NaR_ to the membrane physiology (Fig. 5*A*). I_NaR_ was detectable in basal SGNs at the end of the first postnatal week (P6-P8), but not in apical cells (Fig. 5*A-C*). Soon after hearing onset (P12-P14), I_NaR_ was detectable in SGNs from both regions but the relative magnitude of I_NaR_ was higher in basal SGNs compared to apical SGNs (6.94 ± 0.82 vs 3.55 ± 0.82%; *n* = 11 for each region). This region-specific difference was no longer apparent in SGNs from three-week-old and three-month-old mice (Fig. 5*C*). Comparison of the current–voltage (*I-V*) relationships for I_NaR_ (Fig. 5*D*) and I_NaP_ (Fig. 5*E*) during this period of development confirmed that these currents increased in amplitude relative to maximum I_NaT_ at each age. In the most mature age group (P80-P90), the mean I_NaR_ was ∼8% of maximum I_NaT_ (SGNs from basal and apical regions pooled), and in individual SGNs this was measured as high as 17%.

### ATX-II maximizes persistent and resurgent Na^+^ current amplitudes in cultured SGNs

The sea anemone toxin ATX-II interacts with the gating of Nav channels, resulting in the modulation of nonclassical Na^+^ currents. ATX-II slows conventional inactivation, and in doing so has been observed to enhance I_NaP_ and/or I_NaR_ in sensorimotor neocortical neurons (Mantegazza et al., 1998), hippocampal neurons (Brand et al., 2000), and dorsal root ganglion neurons (Klinger et al., 2012). In the case of I_NaR_, slowing conventional inactivation is suggested to enable the C terminus of Navβ4 to achieve open channel block of the α-subunit during a strong depolarization. To compare the effects of this toxin on primary auditory neurons, we next examined whether ATX-II could enhance I_NaP_ and I_NaR_ in cultured SGNs held under voltage clamp, and then assessed how enhancement of the currents would affect the responses of SGNs under current clamp.

**Figure 6.**
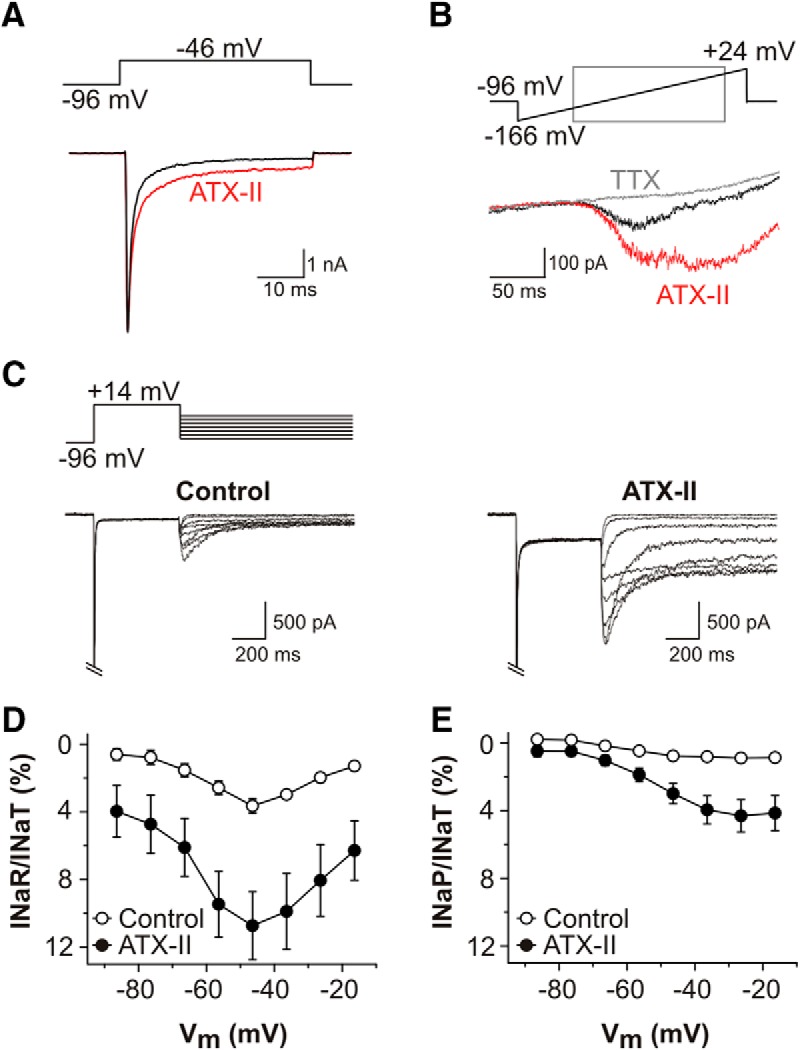
Persistent and resurgent Na^+^ currents are augmented by ATX-II. ***A***, TTX-sensitive current responses evoked by depolarizing voltage step protocol (shown above) in a representative P14 SGN, before (black trace) and after bath application of 5 nM ATX-II (red trace). ATX-II slowed the decay kinetics of I_NaT_. ***B***, I_NaP_ elicited during a voltage ramp protocol in the absence of TTX (black trace) and following ATX-II application (red trace). The additional I_NaP_ activated by ATX-II was blocked by coapplied 100 nM TTX (gray trace). ***C***, TTX-sensitive difference currents in a P14 SGN in response to voltage clamp protocol shown above, before (control, left) and after 5 nM ATX-II (right). I_NaT_ cropped for clarity. ***D***, Mean I_NaR_/I_NaT_ ratio plotted as a function of test potential for five P12-P14 SGNs before (open circles) and after 5 nM ATX-II (filled circles). ***E***, Mean I_NaP_/I_NaT_ ratio plotted as a function of test potentials, before (open circles) and after 5 nM ATX-II (filled circles) for the same SGNs as in ***D***.

In voltage-clamped P12-P14 SGNs (using Na^+^ current isolation conditions) bath application of 5 nM ATX-II did not affect the maximum amplitude of I_NaT_ during 50 ms depolarizing steps from −96 to −46 mV but there was a slowing of its inactivation (Fig. 6*A*). The apparent magnitude of I_NaP_ increased, an effect which was evident at the end of the depolarizing step in the presence of ATX-II. The enhancement of I_NaP_ could also be demonstrated using a voltage ramp paradigm (Fig. 6*B*), and this additional current was blocked by coapplied TTX. During the repolarizing pulse protocol, ATX-II enhanced the I_NaR_/I_NaT_ ratio by approximately three-fold (at −46 mV, control = 3.65 ± 0.43%, ATX-II = 10.73 ± 2.01%, *n* = 5; Fig. 6*C*,*D*), and the I_NaP_/I_NaT_ ratio by approximately five-fold (at −26 mV, control = 0.89 ± 0.15%, ATX-II = 4.31 ± 0.95%, *n* = 5; Fig. 6*C*,*E*).

**Figure 7.**
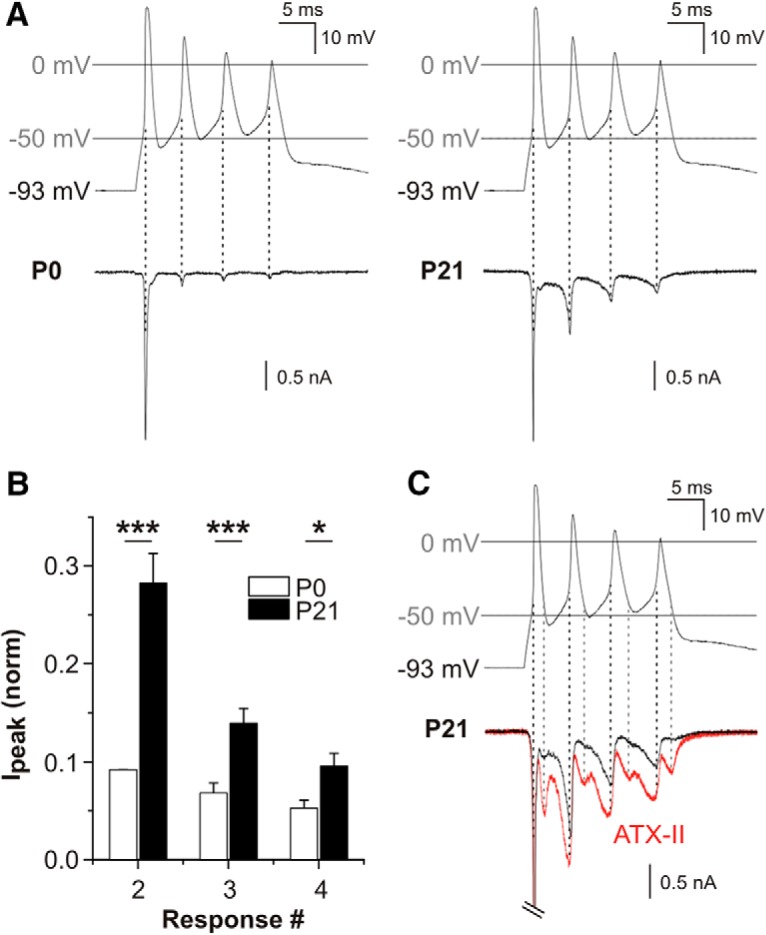
Na^+^ currents elicited by action potential clamp waveforms. ***A***, Na^+^ current responses from a representative P0 SGN (left panel) and a representative P21 SGN (right panel), using a conglomerate action potential as the voltage command stimulus (displayed in upper panels). The action potential wave form was recorded previously from a P21 SGN under current clamp. Isolated Na^+^ currents were recorded using Cs^+^-filled patch electrodes and an external medium supplemented with channel blockers (see Materials and Methods). In response to the depolarizing phase of the first spike in the wave form, the P0 and P21 SGNs responded with transient inward currents of comparable amplitude. In comparison to the P0 SGN, the P21 SGN was able to generate larger amplitude currents in response to the depolarizing phase of the subsequent three spikes. The peak of the inward currents corresponded to the depolarization phase of the individual spikes within the conglomerate wave form (as denoted by discontinuous black line). ***B***, Mean peak currents in response to each spike of the conglomerate action potential, normalized to the first response (*p < 0.05, ***p < 0.001; *n* = 5). ***C***, Current responses in a P21 SGN elicited by the conglomerate action potential wave form, before (black trace) and after 5 nM ATX-II application (red trace). The response to the first spike (cropped for clarity of subsequent responses) was unchanged after ATX-II. Subsequent responses to the depolarizing phases of command spikes were larger following ATX-II application (denoted by discontinuous black line). After ATX-II, there were augmented inward current responses elicited during the repolarization phase of each spike within the conglomerate wave form (denoted by discontinuous gray line).

We next sought to simulate the membrane currents flowing through Na^+^ channels during action potentials in the cultured SGNs, to predict how they might contribute to rapid firing behavior in the auditory nerve. To do this we employed the “action potential clamp” technique which was used originally to record I_NaR_ in cerebellar Purkinje neurons (Raman and Bean, 1997). For the experiments in Figure 7, a “conglomerate” action potential response was recorded previously in a P21 SGN under current clamp mode, and this was used subsequently as the command wave form to be applied to voltage clamped cells. This technique allows ion channels to experience the same trajectory of voltage as during a naturally occurring action potential, and thus any isolated TTX-sensitive Na^+^ current flows with a similar time course and size as during spiking behavior (Bean, 2007).

In P0 SGNs (i.e., before I_NaR_ is observed; Fig. 5*B*), the action potential clamp elicited a rapidly-inactivating inward current that coincided with the depolarizing phase of the first spike (Fig. 7*A*). The depolarization phases of subsequent spikes coincided with relatively smaller evoked inward currents (i.e., responses 2-4). The same procedure applied to P21 SGNs elicited a first inactivating inward current that was comparable in amplitude to that in P0 SGNs. However, the currents evoked by the depolarizing phase of the subsequent spikes were all larger in comparison to those in P0 cells (Fig. 7*B*; ANOVA, *F*_(1,24)_ = 123.36, *p* = 6.09 × 10^−11^). The effect of age was significant across all the individual responses (*post hoc* Bonferroni tests: response 2: 27.7 ± 2.3 vs 8.1 ± 0.7%, *p* = 1.33 × 10^−9^; response 3: 14.1 ± 1.1 vs 5.1 ± 0.8%, *p* = 0.0006; response 4: 9.7 ± 1.0 vs 3.7 ± 0.7, *p* = 0.043; *n* = 5). Throughout the action potential clamp wave form there was a small maintained inward current component in P21 SGNs, which most likely reflected I_NaP_ activation. In all cases at P0, however, there was no inward current evident between the evoked responses. This observation suggested that there was no I_NaP_ at this age, perhaps consistent with the lack of I_NaR_ in SGNs at this developmental stage (Fig. 5*B*).

The ability of P21 SGNs to generate larger currents in response to the repetitive command spikes suggested a decreased refractoriness, possibly enabled by I_NaR_ flowing through the pool of noninactivated channels between command spikes (Bean, 2007). To examine the currents flowing during the repolarization phase more closely, the action potential clamp was applied to P21 SGNs before and after bath application of 5 nM ATX-II (Fig. 7*C*). Consistent with our previous observations of the lack of an effect of ATX-II on I_NaT_ maximum amplitude, there was no appreciable effect on the amplitude of the inward current response coinciding with the first depolarizing phase of the command wave form, but in the presence of ATX-II there were larger currents elicited by the depolarization phase of the subsequent command spikes. This suggests that the P21 SGNs were more able to respond to repetitive stimuli once I_NaP_ and/or I_NaR_ were augmented by ATX-II. Importantly, ATX-II also enhanced the separate inward currents that flowed during the repolarization phases of the command action potentials (Fig. 7*C*, discontinuous gray lines). In all cases, these inward currents were larger following drug exposure. Together, these results suggest that I_NaP_ and I_NaR_ are activated during action potentials in SGNs, and that this raises the likelihood of rapid firing in the auditory nerve.

**Figure 8.**
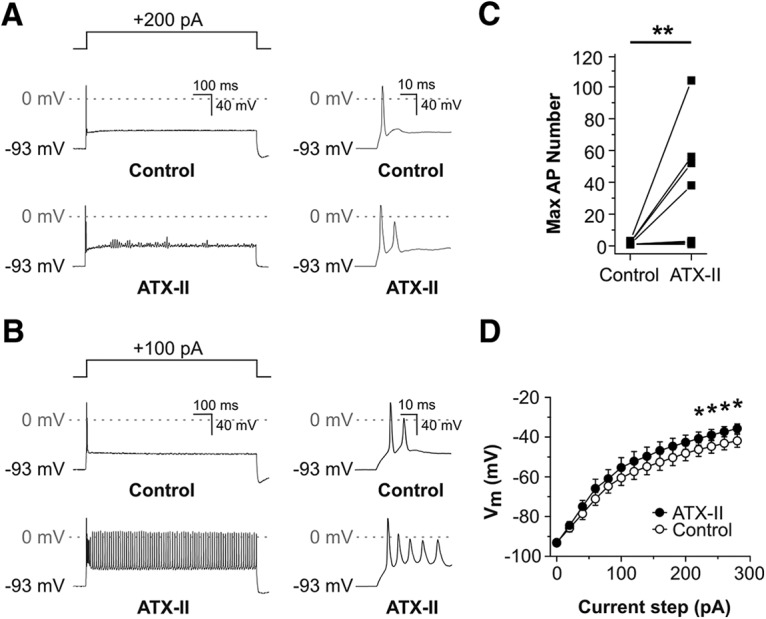
ATX-II application enhanced the excitability of posthearing onset SGNs during current clamp recordings. ***A***, Voltage response from a rapidly-adapting P14 SGN during a sustained depolarizing current injection (shown in upper panel), before (control, middle panel), and after (lower panel) 5 nM ATX-II application. ATX-II application resulted in a slowing of adaptation (inset, right) and oscillation of the membrane potential, typically around −40 mV. ***B***, In another P14 SGN, ATX-II application resulted in a loss of adaptation during sustained depolarizing current injection. ***C***, Effect of 5 nM ATX-II on the maximum action potential (AP) number during 800-ms depolarizing current injections in nine rapidly-adapting SGNs (**p < 0.01; *n* = 9). ***D***, Comparison of the mean voltage-current relationship before and after 5 nM ATX-II. Steady-state voltage measured 10 ms from the end of 800-ms current injections applied in 20-pA increments from a holding potential of −93 mV. ATX-II induced a significant depolarization of the membrane potential for current steps >200 pA (*p < 0.05; *n* = 9).

**Figure 9.**
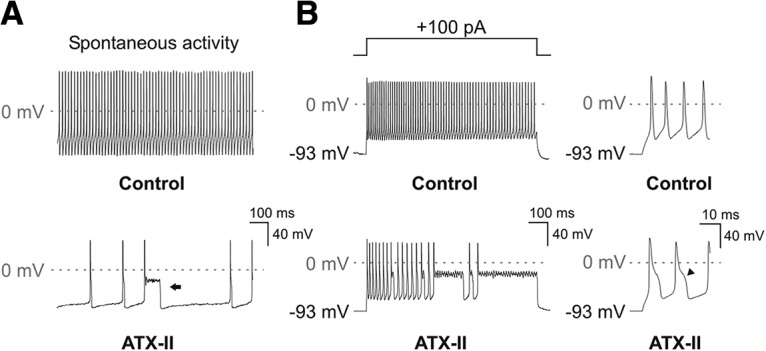
ATX-II induces metastable episodes of the membrane potential and broadens action potentials in nonadapting SGNs. ***A***, During a current clamp recording under control conditions (upper panel), a P14 SGN generated action potentials spontaneously without current injection. Following the bath application of 5 nM ATX-II (lower panel), the SGN adopted a slower spontaneous firing behavior, with broader action potentials interspersed with long-lasting depolarizing events (arrow). ***B***, In the same SGN, a depolarizing current injection (shown in upper left panel) activated a nonadapting response (middle left panel). After application of 5 nM ATX-II, the repetitive action potentials became interspersed by metastable events of variable lengths (lower left panel). Individual action potentials became broader after ATX-II (lower right panel), each with a short-lasting shoulder during the repolarization phase (arrowheads).

To examine the effects of ATX-II on excitability and firing behavior, current clamp experiments were conducted on SGNs cultured from hearing mice (P12-P14; Figs. 8, 9). Bath application of ATX-II had the effects of increasing membrane excitability and decreasing the membrane resistance, observations consistent with the potentiation of I_NaP_ and I_NaR_. A subset of SGNs display rapidly-adapting behavior in response to square-wave current injections in various preparations under control conditions, generating only a few action potentials at the beginning of the stimulus (Santos-Sacchi, 1993; Jagger and Housley, 2002; Szabó et al., 2002; Jagger and Housley, 2003; Rutherford et al., 2012; Crozier and Davis, 2014; Ballestero et al., 2015; Smith et al., 2015). In rapidly-adapting SGNs, following bath application of 5 nM ATX-II additional action potentials were elicited during the current injection (Fig. 8*A-C*). In 4/9 rapidly-adapting SGNs, ATX-II had the effect of changing the firing behavior into a nonadapting response (Fig. 8*B*). The application of ATX-II resulted in a significant increase in the median action potential number (Fig. 8*C*, paired Wilcoxon signed rank test, z = −2.46, *p* = 0.0078, *n* = 9). ATX-II significantly increased the steady-state voltage in response to current injection (Fig. 8*D*; Bonferroni-adjusted paired *t* test; current step 220 pA *p* = 0.042; 240 pA *p* = 0.032; 260 pA *p* = 0.027; 280 pA *p* = 0.018). Together, these effects of ATX-II further support the hypothesis that I_NaR_ and I_NaP_ can increase the excitability of rapidly-adapting SGNs.

Some SGNs cultured from hearing mice respond to maintained depolarizing inputs with a nonadapting response, and some of these cells fire spontaneously at their resting potential (Wang et al., 2013; Smith et al., 2015). These nonadapting SGNs generally have a lower amount of LVA K^+^ current compared to their rapidly-adapting counterparts. We used ATX-II to examine the effects of maximizing I_NaR_ and I_NaP_ on the distinctive firing behavior of these neurons. In SGNs that fired spontaneously at their resting potential under control conditions (Fig. 9*A*, upper panel), bath application of 5 nM ATX-II had the unexpected effect of slowing the spontaneous activity (Fig. 9*A*, lower panel; control 65 Hz, ATX-II 8 Hz, measured in the amplifier’s *I* = 0 mode). Following ATX-II application, occasional action potentials triggered long-lasting depolarizing shifts of the membrane potential that typically lasted 50-100 ms, and these shifts had the effect of preventing regenerative firing. These depolarizing episodes resembled a “metastable condition” induced by ATX-II in regular spiking neocortical pyramidal neurons (Mantegazza et al., 1998), a phenomenon ascribed to the maximal activation of I_NaP_.

In nonadapting SGNs under control conditions, depolarizing current injections activated trains of regularly shaped action potentials (Fig. 9*B*, upper panel). Bath application of 5 nM ATX-II again slowed the firing rate during these evoked responses (Fig. 9*B*, lower panel; control 80 Hz, ATX-II 40 Hz, measured during a +100 pA current injection in current clamp mode), and also resulted in metastable episodes (4/4 nonadapting SGNs). As before, these events had the effect of preventing regenerative firing. Close examination revealed a broadening of individual action potentials following ATX-II application, with the introduction of a distinct short-lasting shoulder during the repolarization phase (Fig. 9*B*, lower right panel).

## Discussion

This study resulted from the serendipitous observation of I_NaP_ in SGNs, a depolarization-activated inward current that would ordinarily be masked by an LVA I_K_ (Smith et al., 2015). Further investigation identified another TTX-sensitive Na^+^ current, namely I_NaR_. To our knowledge this is the first description of these nonclassical Na^+^ currents in the mammalian auditory periphery. The unusual conditions required to reveal I_NaP_ and I_NaR_ may explain why they have evaded previous description in SGNs. LVA I_K_, I_Ca_, and I_h_ must all be blocked, as they activate over the same voltage range as I_NaP_. Similarly, I_NaR_ is only revealed during repolarizing pulses after strong depolarization, at membrane potentials where HVA I_K_ activates. Furthermore, the late appearance of these currents during auditory development means they would not be observed in recordings from neonatal SGNs. We predict that under normal circumstances (1) small depolarizations of the first heminode would activate the noninactivating subthreshold I_NaP_ which would bring the local membrane potential nearer to its threshold for firing and so increase the effectiveness of subsequent inputs, and (2) the open channel block mediated by Navβ4 subunits would increase the number of noninactivated Na^+^ channels, allowing I_NaR_ to flow during the repolarization phase. This would prevent a deep hyperpolarization between action potentials, thus supporting the generation of a new spike and enabling high-frequency firing.

### Persistent and resurgent Na^+^ currents in cultured SGNs

For this study we employed a preparation of dissociated SGNs from the cochleae of mice, from the early postnatal period until around three months of age. This approach has been used widely over a number of years, and has led to the discovery of numerous ion channel mechanisms that have enhanced our fundamental understanding of auditory coding in the mature cochlea (Rusznák and Szucs, 2009). This preparation has several advantages, including the ease of access to the neuronal soma which can only occur following the retraction of endogenous glia and myelin *in vitro*, and the ability to record from adult neurons which is precluded in slice preparations by the advance of ossification (Jagger and Housley, 2003). However, both the potential damage induced by the process of dissociation and the artificial conditions imposed on neurons in culture must be considered when interpreting any findings. Survival rates of SGNs may be low (Vieira et al., 2007), with the remaining cells representing only a subpopulation which might influence the conclusions drawn. There is evidence that time in culture can affect ion channel expression and the resulting firing characteristics of inner ear ganglion neurons (Liu et al., 2016; Cai et al., 2017). It is important therefore, that combinations of techniques are used to qualify observed electrophysiological mechanisms, such as confirmation of ion channel expression and localization using antibody detection methods.

Our experiments using SGNs cultured from hearing mice revealed I_NaP_ as a sustained inward current elicited during depolarizing voltage ramps or voltage steps. Our data suggest I_NaP_ has a hyperpolarized activation compared to that of the classical I_NaT_. The steady-state activation curve for the noninactivating Na^+^ conductance (G_NaP_) was half activated around −55 mV, which is comparable to values determined in neurons from the hippocampus (French et al., 1990) and sensorimotor cortex (Brown et al., 1994). A persistent current has been described previously in SGNs acutely dissociated from guinea pigs (Santos-Sacchi, 1993). This noninactivating “slow inward current” activated around −70 mV and was also TTX-sensitive, but it had a reversal potential that suggested it was mediated by multiple ionic species. It was blocked by 50 µM Cd^2+^, a concentration below that used here in our Na^+^ current isolation solution (300 µM). The relationship between the current in guinea pig SGNs and the I_NaP_ we have described in mouse SGNs may require further study.

Voltage step or action potential clamp paradigms revealed I_NaR_ as an inward current activating during repolarization following strong depolarization. The activation voltage and kinetics of I_NaR_ were comparable to those described first in cerebellar Purkinje neurons (Raman and Bean, 1997). These characteristics support the hypothesis that I_NaR_ acts to minimize the refractory period, when channels mediating I_NaT_ would otherwise be inactivated. Evidence suggests that the requisite channel subunits for I_NaR_ are present in SGNs. The effects of a Nav1.6-specific toxin, and the localization of Nav1.6 immunofluorescence seen here and by others (Hossain et al., 2005; Kim and Rutherford, 2016), suggest that this subunit may mediate all three currents. Nav1.1 subunits, which can mediate I_NaR_ under certain circumstances (Lewis and Raman, 2014), have been detected within the first heminode of SGNs in the rat cochlea, although not within their nodes of Ranvier (Kim and Rutherford, 2016). Localization of Navβ4 subunits within the spike generator region and nodes of Ranvier in the spiral ganglion are consistent with models for I_NaR_ in other fast-spiking neurons (Cruz et al., 2011; Theile and Cummins, 2011; Lewis and Raman, 2014).

The effects of ATX-II on I_NaP_ and I_NaR_, and the resulting changes of SGN firing properties were reminiscent of those seen in other neuronal types (Mantegazza et al., 1998; Brand et al., 2000; Klinger et al., 2012). This further suggests that the currents we identified in SGNs share a common biophysical basis with I_NaP_ and I_NaR_ described elsewhere. Maximal enhancements of I_NaP_ and I_NaR_ had differential effects on the excitability of rapidly-adapting and nonadapting SGNs. Rapid adaptation in SGNs has been linked to the expression of activated Kv1-type channels, which prevent repetitive firing during sustained current injections (Szabó et al., 2002; Wang et al., 2013; Crozier and Davis, 2014; Smith et al., 2015). Application of ATX-II to rapidly-adapting cells was associated with an increased likelihood of firing additional action potentials, suggesting that the depolarizing effect of maximally enhancing I_NaP_ and I_NaR_ was sufficient to overcome the inhibitory influence of the Kv1-mediated currents. In the nonadapting cells, which have minimal Kv1-mediated currents, ATX-II had the effect of broadening action potentials and even inducing metastable depolarizations. This resembled the effects of ATX-II via increased I_NaP_ in neocortical pyramidal neurons (Mantegazza et al., 1998), and the sustained depolarizations probably reflect a lack of sufficient repolarization by K^+^ channels. Future studies should determine how I_NaP_ and I_NaR_ contribute to SGN responses during more physiologic stimuli, preferably in intact cochlear preparations.

### Rapid spiking behavior in the auditory pathway

SGNs in the auditory nerve are among the fastest spiking single units in the nervous system (Heil and Peterson, 2015). They are able to achieve a high temporal precision which preserves the auditory code generated by the presynaptic specializations of IHCs (Rutherford et al., 2012). It is perhaps not surprising, therefore, that SGNs employ mechanisms common to other fast-spiking neurons. By providing subthreshold depolarizing drive (in the case of I_NaP_), or by ensuring there are pools of noninactivated Na^+^ channels available immediately following action potential depolarization (in the case of I_NaR_), these currents may contribute to the regulation of spontaneous and evoked firing rates in the auditory periphery. I_NaP_ and I_NaR_ have been observed in the medial nucleus of the trapezoid body (Leão et al., 2006; Huang and Trussell, 2008; Kim et al., 2010; Berret et al., 2016), an auditory center renowned for its preservation of temporal fidelity. Also, I_NaP_ supports burst firing in primary afferents of the goldfish sacculus, which in turn amplifies resonance in their target Mauthner neurons (Curti et al., 2008).

Spontaneous activity may contribute to the development of normal mammalian hearing. Hearing onset in altricial mammals such as cats and rodents occurs during the second postnatal week. Before this, low-frequency spontaneous firing rates (SFRs, typically <5 spikes/s) are seen in auditory nerve recordings from postnatal kittens (Jones et al., 2007), and in loose patch recordings from rat SGNs in cochlear explants (Tritsch and Bergles, 2010). This spontaneous activity may contribute to the establishment of tonotopic maps in the auditory brain (Jones et al., 2007). A recent *in vitro* study reported that just after hearing onset in rats (P15-P17), SGNs have SFRs ranging from 0–16 spikes/s (Wu et al., 2016). This range diversifies rapidly over the following weeks (P19-P21, 0–44 spikes/s; P29-P32, 0–55 spikes/s), and refractory periods shorten accordingly. Our observation of increasing I_NaP_ and I_NaR_ during this period may help to explain these changes. As there were no obvious regional differences in I_NaP_ and I_NaR_ in the SGNs cultured from animals aged P21 and older, we suggest that those observed in the younger animals were purely developmental, and reflected a progressive maturation of cell properties (including concomitant increases of K^+^ current amplitudes) from the cochlear base toward the apex.

The rapid spontaneous and evoked spiking observed in mature SGNs points to tightly controlled refractoriness. For type I afferents, the mean absolute refractory period (during which no second spike can be evoked) has been measured as 0.33 ms, and the time constant of the mean relative refractory period (during which the probability of a spike increases with time, or the stimulus strength required to trigger a spike with a given probability decreases with time) has been measured as 0.41 ms (Miller et al., 2001), which are brief even in comparison to rapid-spiking neurons such as cerebellar Purkinje neurons (Khaliq and Raman, 2005). In the auditory nerve there is a high density of Nav channel subunits at the first heminode, the site of spike initiation, and also at nodes of Ranvier (Hossain et al., 2005; Smith et al., 2015; Kim and Rutherford, 2016). This organization first appears between P5-P7 in rats (Kim and Rutherford, 2016), coinciding with increased maximal firing rates (Tritsch and Bergles, 2010). This period is roughly equivalent to that in mice, when we first observed I_NaP_ and I_NaR_. By P20-P21, in the rat, there is coexpression of Nav1.1 and Nav1.6 channels at the first heminode, which can both mediate I_NaR_, but only Nav1.6 is detected at subsequent nodes of Ranvier (Kim and Rutherford, 2016). In the young adult mouse there is a comparable distribution of Nav1.6 immunofluorescence, as shown here and elsewhere (Hossain et al., 2005), and this pattern resembles that of Kv3.1b subunits (Smith et al., 2015), whose activation and deactivation kinetics enable the ultra-rapid repolarization essential for repetitive firing. Together, these observations suggest the existence of mechanisms ensuring rapid recovery from action potential firing in SGNs.

### Persistent and resurgent Na^+^ currents in health and disease

There is an increasing appreciation of how I_NaP_ and I_NaR_ contribute to normal spontaneous and evoked firing. I_NaP_ typically activates at subthreshold membrane potentials, a characteristic that implicates it in boosting distal synaptic potentials to reach the soma (Crill, 1996), and in repetitive action potential generation at the axon initial segment (Osorio et al., 2010). I_NaR_ contributes to normal function in ∼20 neuronal types, the best studied of these being cerebellar Purkinje neurons (Raman and Bean, 1997; Raman et al., 1997; Grieco et al., 2005; Akemann and Knöpfel, 2006; Yan et al., 2014; Ransdell et al., 2017). These inhibitory neurons have SFRs of ∼30–50 spikes/s and can support evoked firing up to ∼250 spikes/s. This ability is attributed to I_NaR_ mediated via Nav1.6/Navβ4 channels localized at the axon initial segment. In Purkinje neurons, the rapid repolarizing action of Kv3 channels complements the function of I_NaR_ (Akemann and Knöpfel, 2006), in a manner comparable to that we propose here for SGNs.

Nav1.6 subunits underlie I_NaP_ and I_NaR_ in various neuronal types, and alterations of Nav1.6 expression or gating characteristics result in disease phenotypes. In Nav1.6-null mice, I_NaP_ is down-regulated in cerebellar Purkinje neurons (Raman et al., 1997; Grieco and Raman, 2004), cortical pyramidal neurons (Maurice et al., 2001) and cerebellar granule cells (Osorio et al., 2010). There is a 90% decrease of I_NaR_ in Purkinje cells of Nav1.6-null mice (Raman and Bean, 1997), suggesting that this subunit underlies the majority of the endogenous current. Both I_NaP_ and I_NaR_ have been implicated in disease pathologies related to altered cellular excitability (Cruz et al., 2011; Theile and Cummins, 2011; O'Brien and Meisler, 2013; Lewis and Raman, 2014). Studies of epilepsy models suggest that alterations of Nav1.6 subunit gating lead to enhanced I_NaR_, resulting in neuronal hyperexcitability and epileptogenesis (Hargus et al., 2013). In dorsal root ganglion neurons the chemotherapy drug oxaliplatin slows inactivation of Nav1.6, which enhances I_NaP_ and I_NaR_, and may cause acute cooling-aggravated neuropathy (Sittl et al., 2012).

Mice with a targeted deletion of Navβ4 in the cerebellum display motor control deficits, and their Purkinje neurons have reduced spontaneous and evoked firing rates (Ransdell et al., 2017). However, the amplitude of I_NaR_ reduces by only ∼50% in this model, suggesting that although Navβ4 contributes to I_NaR_ generation additional mechanisms may be required. I_NaP_ and I_NaR_ amplitudes can be decreased by RNA interference techniques (Bant and Raman, 2010; Xie et al., 2013; Yan et al., 2014; Ransdell et al., 2017), suggesting that specific *in vivo* targeting of the channels mediating these currents is a viable therapeutic option for conditions of neuronal hyperexcitability. Accordingly, these channel subunits may represent novel targets for treating neuropathies in the auditory periphery. Future studies should further delineate their contribution to normal hearing, and identify how each current has site-specific actions contributing to fast spiking in the mature auditory nerve.

## Synthesis

Reviewing Editor: Alfonso Araque, University of Minnesota Decisions are customarily a result of the Reviewing Editor and the peer reviewers coming together and discussing their recommendations until a consensus is reached. When revisions are invited, a fact-based synthesis statement explaining their decision and outlining what is needed to prepare a revision will be listed below. The following reviewer(s) agreed to reveal their identity: Radha Kalluri, David Furness.

While both reviewers are in general satisfied with the responses, one of the reviewers has few minor suggestions. In particular, including a brief discussion on pros and cons of using cell cultures as experimental model. There are also some minor points about missing data description. I believe that addressing these minor issues will enhance the strength of the manuscript.

I am looking forward to receiving the final revised version of the manuscript.

Sincerely

Specific comments of the reviewers:

Reviewer 1.

The authors have carefully addressed the principle concerns of a previous round of reviews to improve the scholarship and functional interpretation of these interesting results. I am satisfied that this manuscript makes a significant contribution to the field.

Reviewer 2.

This is very interesting manuscript that clearly adds knowledge to the field by moving towards an explanation of the temporal properties of SGNs in relation to the need for rapid firing by afferent auditory nerve fibres. Although I am not primarily an electrophysiologist, I can see that the data are robust and supportive of the authors' conclusions. The arguments in the Discussion are well founded and reasonably easy to follow.

The authors have responded appropriately to previous reviews and their rebuttal justifies the current version of the MS. However, I feel that the MS needs some further alteration in two areas:

1. The culturing of the SGNs is potentially going to change their properties as has been mentioned by one of the previous reviewers of this article. I feel that in places (e.g. abstract lines 28 - 30; introduction lines 92 - 93) the significance of this is underplayed. It is not a fundamental flaw in the study, but a potential confounding factor. For example, survival rate in SGN cultures is quite low (4%) in one study (Vieira, et al., 2007). This may mean that there is selective loss of subtypes of SGN that are more susceptible. This could influence the conclusions drawn about these cells in culture.

2. The results section makes no mention that I can find about the data relating to Kv1.2 or Crispr as illustrated in Fig. 4. Please make some mention of what this figure shows in relation to these proteins. Finally, what is the blue colour in Fig 4?

Line 325 - 326. Omission of primary antibodies is a poor control; the authors should indicate why they didn't use KO tissue (I assume it is unavailable/lethal).

Minor typographical errors

Line 70: contributes

Line 310: morphology of SGNs

Line 500: the term ‘data’ is plural (datum is singular). Thus this should say ‘data suggest’
